# AS1842856 Reduces β‐Amyloid Burden via Inhibiting PLA2G4A‐Mediated Lysosomal Dysfunction in APP/PS1 Mice

**DOI:** 10.1002/cns.70910

**Published:** 2026-04-28

**Authors:** Da‐Long He, Zheng Wu, Rong‐Jun Jia, Ting‐Yao Wu, Yi‐Min Qiu, Yong‐Gang Fan

**Affiliations:** ^1^ Key Laboratory of Tropical Translational Medicine of Ministry of Education, College of Basic Medical Sciences Hainan Medical University Haikou China; ^2^ Key Laboratory of Medical Cell Biology of Ministry of Education, Key Laboratory of Major Chronic Diseases of Nervous System of Liaoning Province Health Sciences Institute of China Medical University Shenyang China; ^3^ First Affiliated Hospital of Jinzhou Medical University Jinzhou China

**Keywords:** Alzheimer's disease, AS1842856, cytosolic phospholipase A2, glycogen synthase kinase‐3, lysosome

## Abstract

**Aims:**

Both cytosolic phospholipase A2 (PLA2G4A)‐induced lysosomal membrane disruption and glycogen synthase kinase‐3*α*/*β* (GSK3*α*/*β*)‐mediated lysosomal dysfunction have been implicated in neurodegeneration, with a potential regulatory relationship between these two pathways. We recently identified AS1842856 (AS) as a suppressor of GSK3*α*/*β*. This study was therefore designed to investigate whether AS mitigates Alzheimer's disease (AD) progression by targeting PLA2G4A to restore lysosomal homeostasis.

**Methods:**

The therapeutic potential of AS was investigated in APP/PS1 mice by analyzing cognitive function, *β*‐amyloid (A*β*) load, and lysosomal integrity, with its mechanism of action further explored in N2a‐sw cells.

**Results:**

AS treatment reduced GSK3*α*/*β* expression in both APP/PS1 mice and N2a‐sw cells. This suppression led to decreased PLA2G4A levels, restoration of lysosomal membrane integrity, and enhanced lysosomal degradation of A*β*. Consequently, AS administration alleviated A*β* burden and improved cognitive function in APP/PS1 mice. Moreover, AS was found to inhibit NF‐κB‐mediated PLA2G4A expression. Knockdown experiments further revealed that reduced GSK3*β*—but not GSK3*α*—reproduced the suppressive effect on PLA2G4A.

**Conclusion:**

Our study identified the GSK3*β*/NF‐κB/PLA2G4A signaling axis as a novel therapeutic target in AD, and AS could inhibit this axis to mitigate A*β* pathology by promoting lysosomal degradation of A*β*.

## Graphical abstract

AS1842856 reduces intracellular levels of GSK3*α*/*β* by binding to them and promoting their exocytosis. This reduction leads to diminished GSK3*β*‐mediated activation of NF‐κB, resulting in decreased transcription of PLA2G4A and reduced lysosomal damage. Ultimately, this enhances lysosomal degradation of A*β*, lowering A*β* load in APP/PS1 mice.

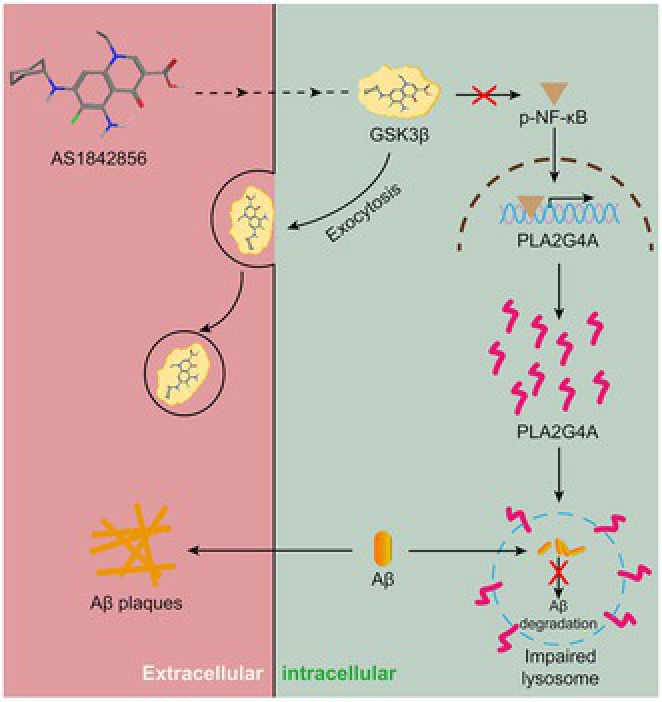

## 1 Introduction

Alzheimer's disease (AD) is the most common neurodegenerative disorder in humans. The main pathological features of AD are senile plaques formed by *β*‐amyloid (A*β*) aggregation and neurofibrillary tangles (NFTs) formed by hyperphosphorylation of Tau protein [1]. Given that A*β* deposition is a central pathogenic mechanism of AD, targeting A*β* in the brain has emerged as a key therapeutic strategy for combating AD [2, 3].

A*β* is generated from the sequential cleavage of *β*‐amyloid precursor protein (APP) by *β*‐secretase and γ‐secretase [4]. The metabolism of A*β* in the brain is primarily orchestrated through the clearance pathway [5, 6]. Several studies revealed that in healthy individuals, the clearance rate of A*β* in the central nervous system is greater than its production rate [7], whereas in the brains of AD patients, A*β* clearance is significantly impaired [8]. Among the various pathways for clearing A*β*, lysosomal degradation stands out as a pivotal mechanism, as lysosomes function as both the cell's degradation center and a signaling hub, playing a key role in cellular homeostasis, development, and aging [9]. Critically, lysosomal acidification in neurons is compromised before extracellular A*β* deposition occurs during AD progression, thereby causing significant A*β* accumulation within dysfunctional lysosomes [10]. The accumulation of A*β* within lysosomes then causes lysosomal membrane damage, increasing lysosomal permeability and ultimately leading to neuronal death [11]. Therefore, improving lysosomal function in neurons is an effective strategy for degrading A*β* and reducing its accumulation in the brain.

Glycogen synthase kinase 3 (GSK3) serves as a key regulator of lysosomal acidification, with pharmacological inhibition of this kinase demonstrating enhanced lysosomal acidification [12]. GSK3 exists as two highly homologous isoforms (GSK3*α* and GSK3*β*) that share conserved structural domains and exhibit similar functions [13]. Accumulating neuropathological evidence reveals a consistent upregulation of both GSK3 isoforms, coupled with cytosolic phospholipase A2 (PLA2G4A), in the brains of AD patients [14, 15, 16, 17]. This co‐occurrence is mechanistically significant, as PLA2G4A ‐mediated lysosomal membrane permeabilization constitutes a critical neurodegenerative pathway [18, 19, 20]. Based on these findings, we propose that GSK3‐mediated lysosomal dysfunction is functionally coupled with PLA2G4A activity—a process by which GSK3*α*/*β* may potentiate PLA2G4A‐driven lysosomal destabilization in the pathogenesis of AD.

AS1842856 (AS) is a small molecule compound identified through affinity screening mass spectrometry technology [21]. Initial studies revealed its effectiveness in alleviating elevated blood glucose levels in type 2 diabetic mice, though its precise mechanism of action remains unclear [21, 22]. In the previous study, we discovered that AS can bind to GSK3*α*/*β* and reduce intracellular levels of GSK3*α*/*β* by promoting their exocytosis, thereby inhibiting Tauopathy in P301S transgenic mice [23]. In this study, we further explored the pharmacological effects of AS in A*β* pathology. We found that AS treatment‐induced downregulation of GSK3*β* inhibits nuclear factor κB (NF‐κB) phosphorylation, which restores lysosomal integrity via inhibiting PLA2G4A transcription, ultimately promoting lysosomal degradation of A*β* and cognitive performance in APP/PS1 transgenic mice. This study indicated that AS may be a potential compound for treating AD.

## 2 Materials and Methods

### 2.1 Cell Lines and Animals

The N2a cells and N2a‐sw cells were a gift from Professor Zhan‐You Wang at China Medical University. 5‐month‐old female C57/BL6 and APP/PS1 (APPswe/PSEN1dE9) mice were purchased from BEIJING HFK BIOSCIENCE Co. LTD. All mice were given free access to food and water and were maintained at constant room temperature in a 12‐h alternating light and dark environment. A total of 12 APP/PS1 mice were randomly divided into two equal groups: vehicle‐treated mice and AS‐treated mice. Mice in the AS group received daily intraperitoneal injections of AS (5 mg/kg; MCE, HY‐100596) for 2 months, and the treatment was continued throughout the behavioral assessments. This study was carried out according to the recommendations of “Laboratory Animals‐Guideline of Welfare and Ethics, The Ethics Committee for Medical Laboratory Animals of China Medical University” (IACUC: No. CMU2023516). The protocol was approved by the Ethics Committee for Medical Laboratory Animals of China Medical University. Other details of behavioral experiment protocols and cell lines treatments can be found in the Supporting Information.

### 2.2 Tissue Collection and Brain Lysate Vesicle Enrichment

Mice were anesthetized via intraperitoneal injection of sodium pentobarbital (50 mg/kg) and then subjected to cardiac perfusion with physiological saline. Following perfusion, the mice were euthanized by decapitation. The brains were rapidly extracted and sectioned sagittally into two halves on an ice pack. One half was preserved in 4% paraformaldehyde for later morphological studies, while the other half was frozen at −80°C for biochemical analysis. To enrich vesicles in brain lysates, the experimental procedure was performed as described in our previous study [23].

### 2.3 Western Blot

The Western blot experimental protocol followed the operational procedures established in our previous studies [23]. In brief, cells or mouse brain tissues were lysed in RIPA (Beyotime Biotechnology, P0013C) containing 1% PMSF (MCE, HY‐B0496), 1% protease inhibitor (MCE, HY‐K0010), and 1% phosphatase inhibitor (MCE, HY‐K0022). The lysate was centrifuged at 10,000 rpm for 20 min at 4°C. The supernatant was collected, and the protein concentration was determined by BCA kit (Beyotime Biotechnology, P0009) according to the manufacturer's instructions. SDS‐PAGE and Tricine‐SDS‐PAGE polyacrylamide gel electrophoresis were performed on 20–40 μg of protein. After electrophoresis, the proteins were transferred onto PVDF membranes (Millipore, IPVH00010), after which the PVDF membranes were blocked with 5% skimmed milk powder for 1 h at room temperature, the primary antibodies was incubated at 4°C overnight. The primary antibodies: rabbit anti‐A*β* (Cell Signaling Technology, 8243, 1:2000), rabbit anti‐APP (Sigma‐Aldrich, A8717, 1:2000) was used to detect C99 and C83, mouse anti‐APP (proteintech, 60342–1‐Ig, 1:5000) was used to detect full length APP (APP‐FL), rabbit anti‐a disintegrin and metalloproteinase 10 (ADAM10, Abcam, Ab1997, 1:1000), mouse anti‐soluble amyloid precursor protein *α* (sAPP*α*, Immuno‐Biological Laboratories, 11088, 1:200), mouse anti‐soluble amyloid precursor protein *β* (sAPP*β*, Immuno‐Biological Laboratories, 10321, 1:500), rabbit anti‐*β*‐site amyloid precursor protein cleaving enzyme 1 (BACE1, ABclonal, A11533, 1:1000), rabbit anti‐Presenilin 1 (PS1, Cell Signaling Technology, 5643S, 1:1000), rabbit anti‐insulin‐degrading enzyme (IDE, ABclonal, A11190, 1:1000), rabbit anti‐apolipoprotein E (ApoE, Immunoway Biotechnology Company, YT0273, 1:500), rabbit anti‐Matrix metallopeptidase 9 (MMP9, ABclonal, A24220, 1:500), rabbit anti‐LDL receptor‐associated protein 1 (LRP1, proteintech, 26106–1‐AP, 1:1000), rabbit anti‐GSK3*α*/*β* (Immunoway Biotechnology Company, YT2081, 1:1000), mouse anti‐Alix (Cell Signaling Technology, 2171, 1:1000), rabbit anti‐ATP6V0A1 (proteintech, 13828–1‐AP, 1:1000), rabbit anti‐lysosome associated protein 1 (LAMP1, proteintech, 21997–1‐AP, 1:1000), rabbit anti‐LAMP2 (proteintech, 27823–1‐AP, 1:1000), rabbit anti‐Galectin‐3 (Gal‐3, Immunoway Biotechnology, YM8817, 1:2000), rabbit anti‐Neprilysin (NEP, proteintech, 18,008–1‐AP, 1:500), rabbit anti‐P62 (proteintech, 18420–1‐AP, 1:500), rabbit anti‐LC3B‐I/II (ABMART Inc., T55992, 1:1000), rabbit anti‐Transcription Factor EB (TFEB, Cell Signaling Technology, 83010, 1:1000), rabbit anti‐p‐TFEB (Cell Signaling Technology, 37681, 1:1000), rabbit anti‐PLA2G4A (Immunoway Biotechnology, YT5176, 1:1000), rabbit anti‐GSK3*α* (Abcam, ab40870, 1:1000), mouse anti‐GSK3*β* (Cell Signaling Technology, 9832, 1:1000), mouse anti‐GFP (ABclonal, AE012, 1:2000), rabbit anti‐p‐GSK3*β* (Cell Signaling Technology, 5558S, 1:1000), rabbit anti‐NF‐κB p65 (Cell Signaling Technology, 8242, 1:1000), rabbit anti‐p‐NF‐κB p65 (Cell Signaling Technology, 3033, 1:1000) and mouse anti‐*β*‐actin (ABclonal, AC004, 1:5000).

### 2.4 Immunohistochemical Staining

Three equally spaced brain sections (approximately 1 mm apart) were used for each group of mice. Immunohistochemistry experimental steps were performed according to the protocol (Boster Biological Technology, SV0002). Sections were incubated with anti‐A*β* antibody (Cell Signaling Technology, 8243, 1:300) at 4°C overnight. Then, the sections were incubated with secondary antibodies (Boster Biological Technology, SV0002) for 1 h at room temperature and the sections were subjected to DAB (Fuzhou Maixin Bio, DAB‐0031). Images were acquired under a microscope (NI‐E, Nikon). The percentage area covered by A*β* plaques in the cortex and hippocampus was quantified using ImageJ software by an investigator blinded to the experimental groups. A total of 3 coronal sections per mouse (bregma −1.0 to −2.5 mm) were analyzed to ensure representative sampling.

### 2.5 X‐34 and Immunofluorescence Staining

Mouse brain sections were washed 3 times in 1 × PBS for 5 min. Then, sections were transferred to buffer (40% ethanol, 60% 1 × TBS, 1:500 vol. 10 N NaOH) containing a concentration of 2 μM X‐34 (Sigma, SML1954) and stained for 20 min. Finally, sections were washed in washing solution (40% ethanol, 60% 1 × TBS).

After X‐34 staining, mouse brain sections were permeabilized with 0.2% Triton X‐100 for 15 min at room temperature. After being blocked with 5% BSA (sigma‐Aldrich, V900933) for 1 h, the sections were incubated with primary antibodies (Rabbit anti‐Iba1, ABclonal, A19776, 1:200; Mouse anti‐glial fibrillary acidic protein, GFAP, Cell Signaling Technology, 3670, 1:400) overnight at 4°C and washed three times with PBS for 10 min each time. The sections were incubated with secondary antibodies (Goat anti‐Mouse‐IgG Alexa 488, Thermo Fisher Scientific, A‐11001, 1:300; Goat anti‐Rabbit‐IgG Alexa 488, Thermo Fisher Scientific, A‐11008, 1:300) at room temperature for 1 h and blocked with an anti‐fluorescence quencher, washed three times with 1 × PBS for 10 min each time, and blocked with an anti‐fluorescence quencher. Finally, images were acquired using a laser confocal microscope (Nikon A1) and quantified using Image J software.

### 2.6 Cathepsin D (CTSD) and Cathepsin B (CTSB) Activity Assay

For the activity assay of CTSD and CTSB in mouse brain tissue, the steps were carried out with reference to the instructions of the kits (Abcam, ab65302, ab65300). In this experiment, 10 mg of mouse cerebral cortex was taken from each sample for the assay, and the fluorescence intensity was collected in a fluorescence enzyme marker (Cytation 5, BioTek) and each sample was quantified and normalized by BCA.

### 2.7 Real‐Time Quantitative Polymerase Chain Reaction (qPCR)

Total RNA from N2a cells was prepared using the RNA Extract kit (Accurate Biology, AG21101). Reverse transcription of RNA to cDNA using Fast King cDNA First Strand Synthesis Kit (Tiangen Biotech, China). qPCR was performed using a Real‐time qPCR kit (Accurate Biology, AG11701) and a Light Cycler 96 system (Roche, Switzerland). The mRNA expression was calculated using ΔΔCt (threshold cycle, Ct) values normalized to *β*‐actin. Sequences of PLA2G4A primers are as follows: forward, 5′‐ATGGTGGGATTCTCTGGTGTGATG‐3′ and reverse, 5′‐TCTCCTCGGGACCTTTCTCTGG‐3′.

### 2.8 Isolation of Nuclei and Cytoplasmic Components

The N2a cells were treated with AS (0.5 μM) and the solvent control for 24 h. The process of isolation of nuclei and cytoplasmic proteins was carried out with reference to the kit instructions (Beyotime Biotechnology, P0028).

### 2.9 Cycloheximide (CHX) Chase Experiments

A total of 5 × 10^5^ N2a‐sw cells were seeded onto 10 cm plates to culture for 24 h. Cells were treated with 0.5 μM AS and/or CHX (20 μM) for various periods (0, 12, 24, 36 and 48 h). Then, the cells were harvested and analyzed by Western blot.

### 2.10 Enzyme‐Linked Immunosorbent Assay (ELISA)

N2a‐sw cells were treated with AS (0.5 μM) or CAY10650 (0.5 μM) for 24 h. The levels of A*β*_1‐40_ and A*β*_1‐42_ in the culture medium were measured using the A*β*_1‐40_ assay kit (JONLOBIO, JL41245) and the A*β*_1‐42_ assay kit (JONLOBIO, JL41255), with procedures following manufacturer instructions. Absorbance values were recorded at 450 nm using a microplate reader.

### 2.11 Chromatin Immunoprecipitation (ChIP)

ChIP experiments were performed using the ChIP Kit (P2080S, Beyotime, China). Brief steps are as follows: Cells were crosslinked with 1% formaldehyde for 10 min, followed by quenching with 1 M glycine. Chromatin was sonicated and immunoprecipitated using anti‐NF‐κB p65 antibody (Cell Signaling Technology, 8242) or control IgG (Cell Signaling Technology, 3900). Protein‐DNA complexes were captured using protein A/G magnetic beads, eluted, and de‐crosslinked. Purified DNA was analyzed via qPCR for the PLA2G4A promoter region. The PLA2G4A gene target region, encoding a 206 bp fragment, was detected using a forward primer (5′‐CCTCCTTAGCTTTTACTTGG‐3′) and a reverse primer (5′‐GGATTCCAACCCAAAGAAAC‐3′) covering positions + 143 to +349 [24].

### 2.12 Statistical Analysis

All the experiments and analyses are conducted with the experimenter blind to drug treatment. The Student's *t*‐test was performed to calculate the level of significance for comparing two groups, while one‐way analysis of variance (ANOVA) was done for comparing more than two groups. All datasets were tested for normal distribution and homogeneity of variance to confirm that non‐parametric testing was not required. In multigroup studies with parametric variables, *post hoc* tests were conducted only if F in ANOVA (or equivalent) achieved the ‘chosen’ necessary level of statistical significance and there was no significant variance in homogeneity. The immunohistochemical analyses were performed on three sections per sample and averaged, and three technical replicates were used for all cellular experiments. All graphs and statistics were done in GraphPad Prism 8. All data sets were tested for outliers using the Grubbs test. All data were expressed as mean ± SEM, and the significance level for all analyses was set at *p* < 0.05.

## 3 Results

### 3.1 AS Treatment Enhances Cognitive Functions in APP/PS1 Transgenic Mice

**FIGURE 1:**
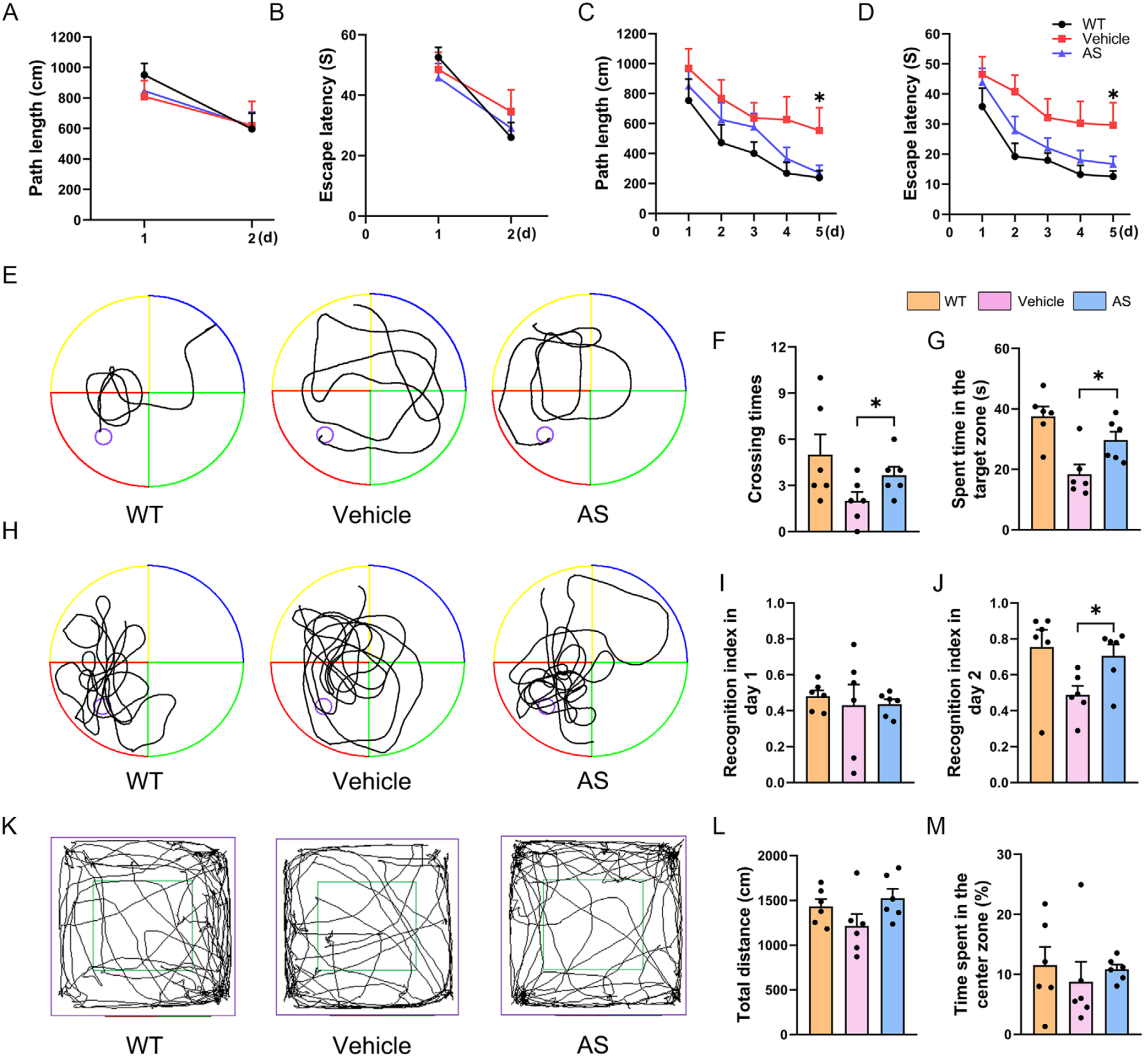
AS treatment enhances cognitive functions in APP/PS1 transgenic mice (A, B) The path lengths and escape latencies to visit the visible platform in the three indicated groups of mice. (C, D) The path lengths and escape latencies to visit the hidden platform in the three indicated groups of mice. (E) The typical path maps of the mice on the fifth day of the hidden platform test. (F) The crossing times in the probe trial test. (G) The time spent in the target zone in the probe trial test. (H) The typical path maps of the mice in the probe trial test. (I‐J) The recognition index on day 1 and day 2. (K) The typical traveling traces of mice during 5 min of open field exploration. (L) The total distances of mice during 5 min of open field exploration. (M) The duration of mice in the center zone. *n* = 6; *p < 0.05.

Morris water maze and new object recognition experiments were performed to investigate the learning and memory ability of mice. The results showed that during the visible platform test, no significant changes were observed in the path lengths (Figure 1A *p* > 0.05) and escape latencies (Figure 1B, *p* > 0.05) in the WT, vehicle‐ and AS‐treated mice, suggesting that there were no significant differences in the motor ability and visual acuity among the three groups of mice. During the hidden platform tests, the vehicle‐treated mice had longer path lengths (Figure 1C,E *p* < 0.05) and escape latencies (Figure 1D,E *p* < 0.05) to reach the platform compared with the AS‐treated mice and WT mice. During the probe trial, AS‐treated mice exhibited a significantly greater number of platform‐site crossovers compared to vehicle‐treated controls (Figure 1F *p* < 0.05), accompanied by an increase in spent time in the quadrant where the platform was located (Figure 1G,H *p* < 0.05). In the new object recognition test, there was no significant difference in the recognition index of the three groups of mice on day 1 (Figure 1I *p* > 0.05), but the AS‐treated mice showed a significant increase in recognition index on day 2 compared with the vehicle‐treated mice (Figure 1J *p* < 0.05). Meanwhile, the open field test showed no significant difference in anxiety‐like behavior among the three groups of mice (Figure 1K–M *p* > 0.05). The above results suggest that AS alleviated cognitive impairment in APP/PS1 transgenic mice.

### 3.2 AS Treatment Decreases A*β* Deposition in the APP/PS1 Mouse Brains

**FIGURE 2:**
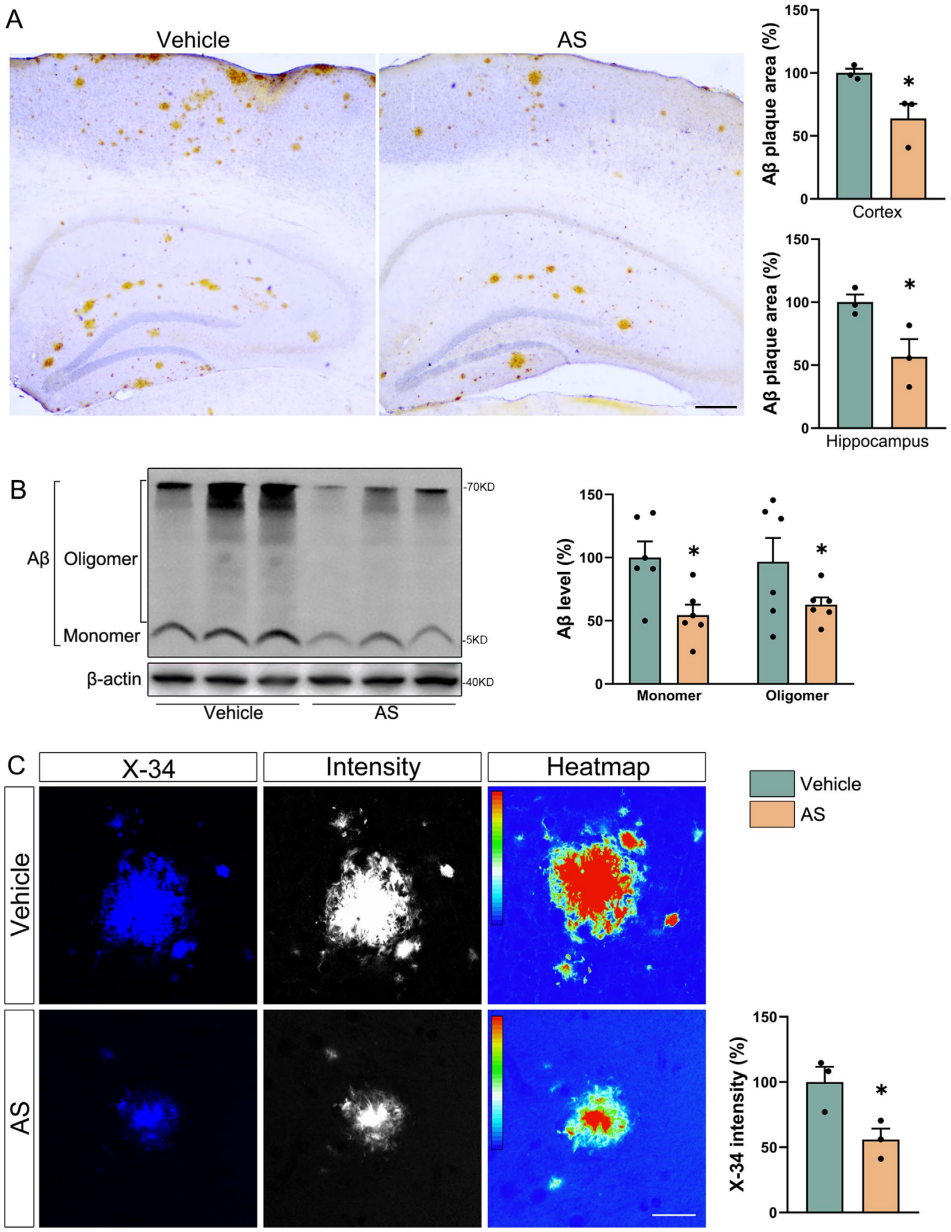
AS treatment decreases A*β* deposition in the APP/PS1 mouse brains. (A) Immunohistochemical staining of A*β*. Scale bar = 200 μm. *n* = 3. (B) Immunoblotting evaluated the expression of A*β* in the cortex of mice. *n* = 6. (C) The X‐34 staining of brain sections from the vehicle and AS‐treated mice. Scale bar = 20 μm. *n* = 3. *p < 0.05.

Given that A*β* deposition is a major pathological feature of APP/PS1 mice, we assessed the effect of AS on A*β* pathology using multiple approaches. Immunohistochemical staining revealed that AS treatment significantly reduced the A*β* plaque area in both the cortex and hippocampus (Figure 2A *p* < 0.05). Consistent with this, immunoblotting analysis confirmed a marked decrease in A*β* content in the brains of AS‐treated mice (Figure 2B *p* < 0.05). Furthermore, X‐34 staining demonstrated that AS treatment also reduced the intensity of A*β* plaque in APP/PS1 mice (Figure 2C *p* < 0.05). Taken together, these results indicate that AS treatment significantly alleviates A*β* deposition in the brains of APP/PS1 transgenic mice.

### 3.3 AS Has no Significant Effect on A*β* Production and Proteins Involved in A*β* Clearance in APP/PS1 Mice

**FIGURE 3:**
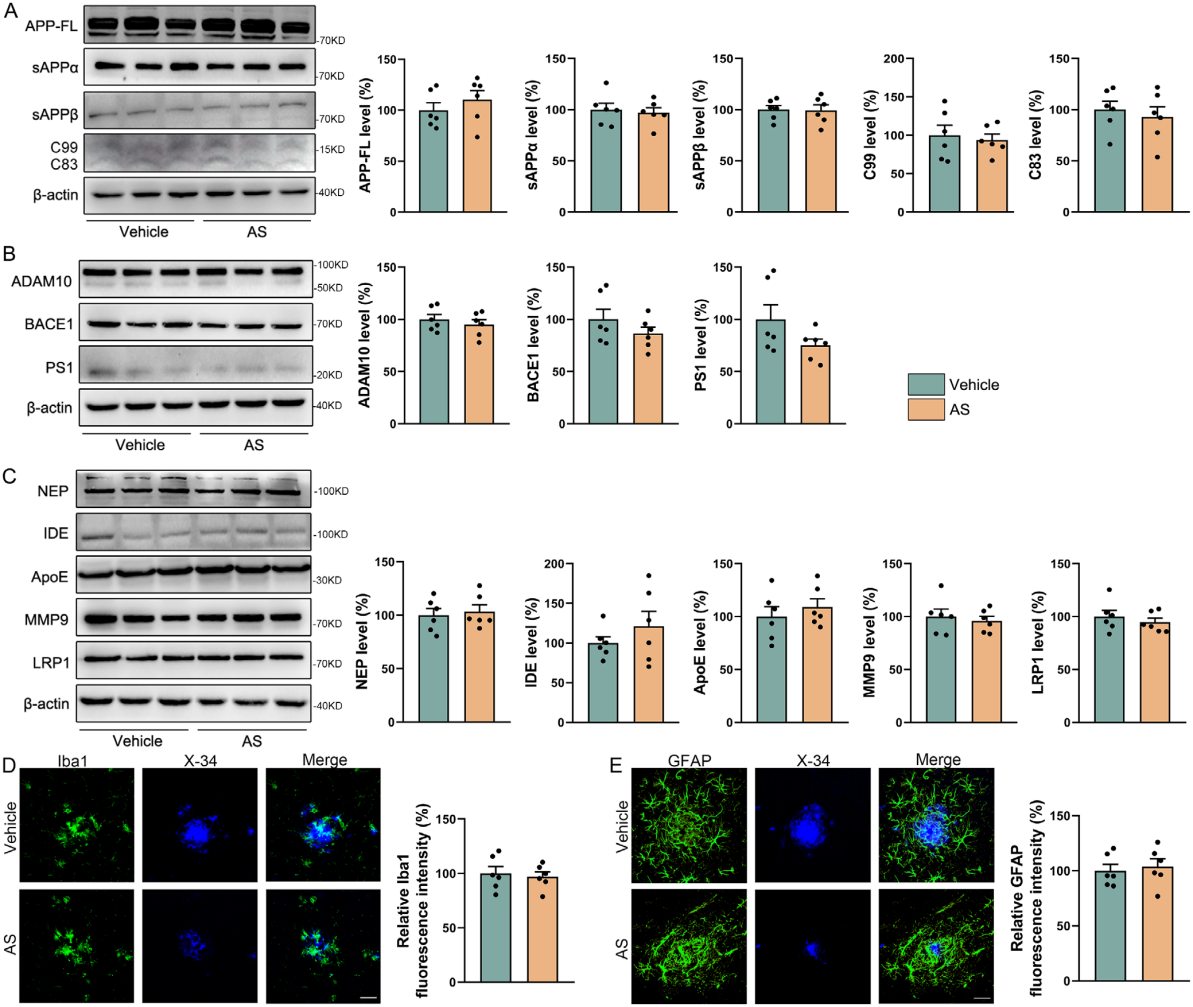
AS has no significant effect on A*β* production and proteins involved in A*β* clearance in APP/PS1 mice. (A, B) Immunoblotting evaluated the expressions of APP‐FL, sAPP*α*, sAPP*β*, C99, C83, ADAM10, BACE1, and PS1 in the cortex of mice. (C) The expressions of NEP, IDE, ApoE, MMP9, and LRP1 in the cortex of mice were evaluated using immunoblotting. (D, E) Immunofluorescence staining shows fluorescence intensity of Iba1 (D), GFAP (E), and its colocalization with X‐34. Scale bar = 20 μm. *n* = 6.

To elucidate the mechanism underlying AS‐mediated reduction of A*β* plaque deposition, we assessed its effects on APP processing and A*β* clearance pathways in APP/PS1 mice. Western blot analysis revealed that AS treatment did not alter the levels of APP‐FL, its *α*/*β*‐secretase cleavage products (C83, C99, sAPP*α* and sAPP*β*), or the related processing enzymes (ADAM10, BACE1 and PS1) (Figure 3A,B *p* > 0.05). Similarly, the expression of key A*β* transport and degradation proteins (NEP, IDE, ApoE, MMP9 and LRP1) remained unaffected (Figure 3C *p* > 0.05). Immunostaining further showed no significant changes in the intensity of A*β* plaque‐associated microglial (Iba1) or astrocytic (GFAP) markers (Figure 3D,E *p* > 0.05). These findings collectively demonstrate that AS does not exert its anti‐A*β* effects through modulating A*β* production or classic clearance pathways.

### 3.4 AS Treatment Improves Lysosomal Function by Reducing PLA2G4A Levels in APP/PS1 Mouse Brains

**FIGURE 4:**
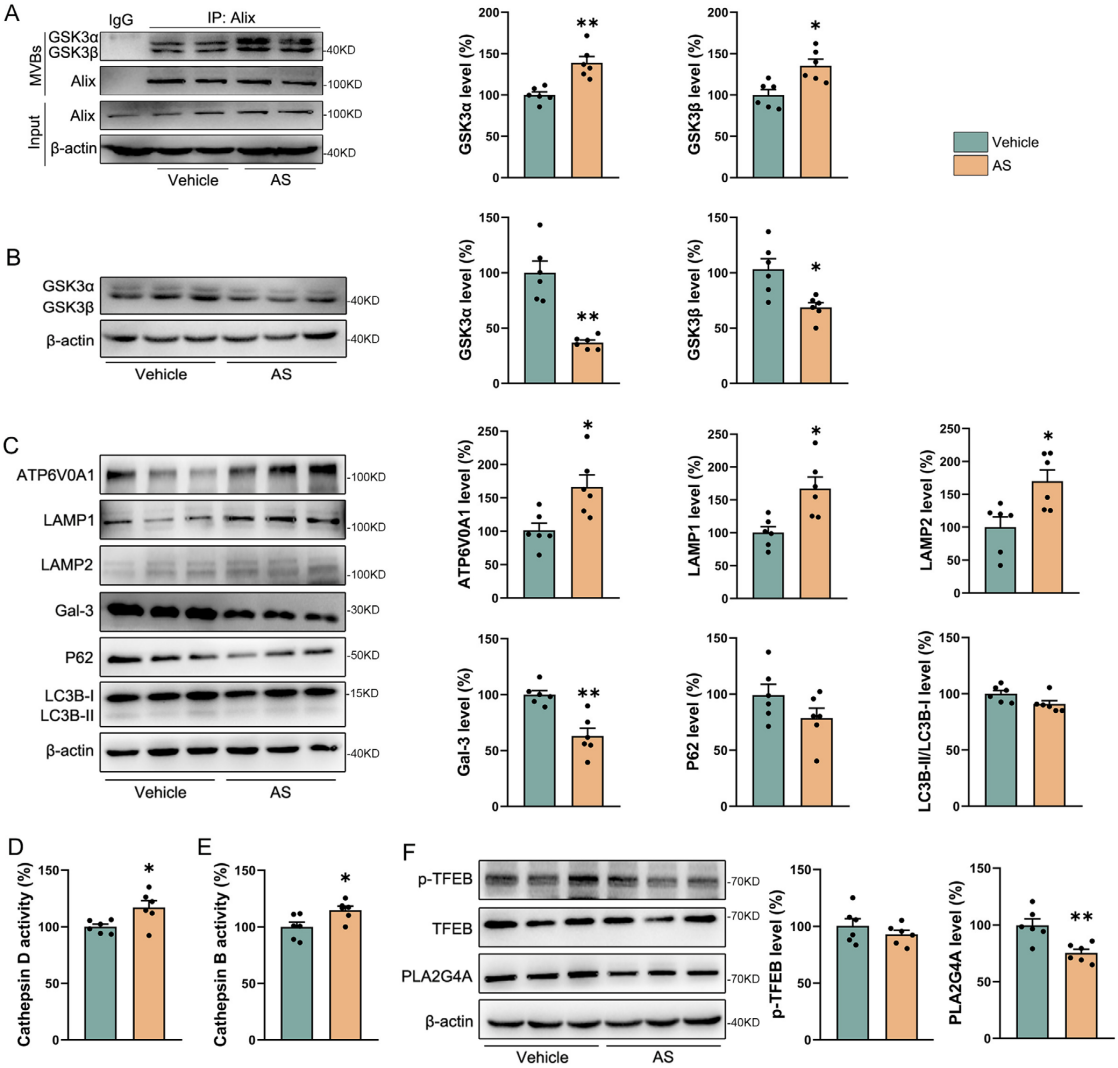
AS treatment improves lysosomal function by reducing PLA2G4A levels in APP/PS1 mouse brains. (A) AS treatment increased the content of GSK3*α*/*β* in the vesicles from the mouse cortex. (B, C) Immunoblotting evaluated the expressions of GSK3*α*/*β*, ATP6V0A1, LAMP, LAMP2, Gal‐3, P62 and LC3B in the cortex of mice. (D, E) Enzyme activities of CTSD (D) and CTSB (E) in cerebral cortex lysates of mice. (F) The expressions of p‐TFEB, TFEB and PLA2G4A in the cortex of mice. *n* = 6; *p < 0.05, **p < 0.01.

Our previous study demonstrated that AS treatment reduces intracellular GSK3*α*/*β* levels in cells and mouse brain tissues by promoting their exocytosis [23]. To determine whether AS decreases GSK3*α*/*β* expression in the brains of APP/PS1 transgenic mice through a similar mechanism, we examined GSK3*α*/*β* content in cortical vesicles. As shown in Figure 4A, AS treatment significantly increased vesicular GSK3*α* (*p* < 0.01) and GSK3*β* (*p* < 0.05) levels in the brains of APP/PS1 mice. Concurrently, AS treatment markedly reduced the expression of GSK3*α* (*p* < 0.01) and GSK3*β* (*p* < 0.05) in brain tissue (Figure 4B), indicating that AS promotes GSK3*α*/*β* exocytosis, thereby lowering its intracellular levels in the APP/PS1 mouse brain.

Given that inhibition of GSK3*α*/*β* has been shown to promote lysosomal acidification [25, 26], while A*β* impairs lysosomal function [27], and considering that lysosomal degradation is a key pathway for A*β* clearance [28], we next investigated whether AS enhances lysosomal function in the brains of APP/PS1 mice. Our results showed that AS treatment upregulated the expression of ATP6V0A1 (*p* < 0.05), lysosome associated protein 1 (LAMP1, *p* < 0.05), and LAMP2 (*p* < 0.05), and reduced the level of the lysosomal damage marker Gal‐3 (*p* < 0.01), without significantly affecting P62 or LC3B expression (*p* > 0.05, Figure 4C). In addition, AS treatment significantly increased the enzymatic activities of CTSD and CTSB in the brain (Figure 4D,E *p* < 0.05), suggesting enhanced lysosomal function.

To explore the mechanisms underlying lysosomal function recovery, we examined key regulators of lysosomal biogenesis and phospholipases involved in lysosomal damage. AS treatment did not significantly alter p‐TFEB levels (Figure 4F *p* > 0.05), but markedly decreased the expression of PLA2G4A (Figure 4F *p* < 0.01), a major brain phospholipase known to disrupt lysosomal membrane integrity [18]. These findings suggest that AS‐induced downregulation of GSK3*α*/*β* enhances lysosomal function by suppressing PLA2G4A expression in the brains of APP/PS1 mice.

### 3.5 AS Treatment Reduces PLA2G4A Level and Ameliorates Lysosomal Impairment In Vitro

**FIGURE 5:**
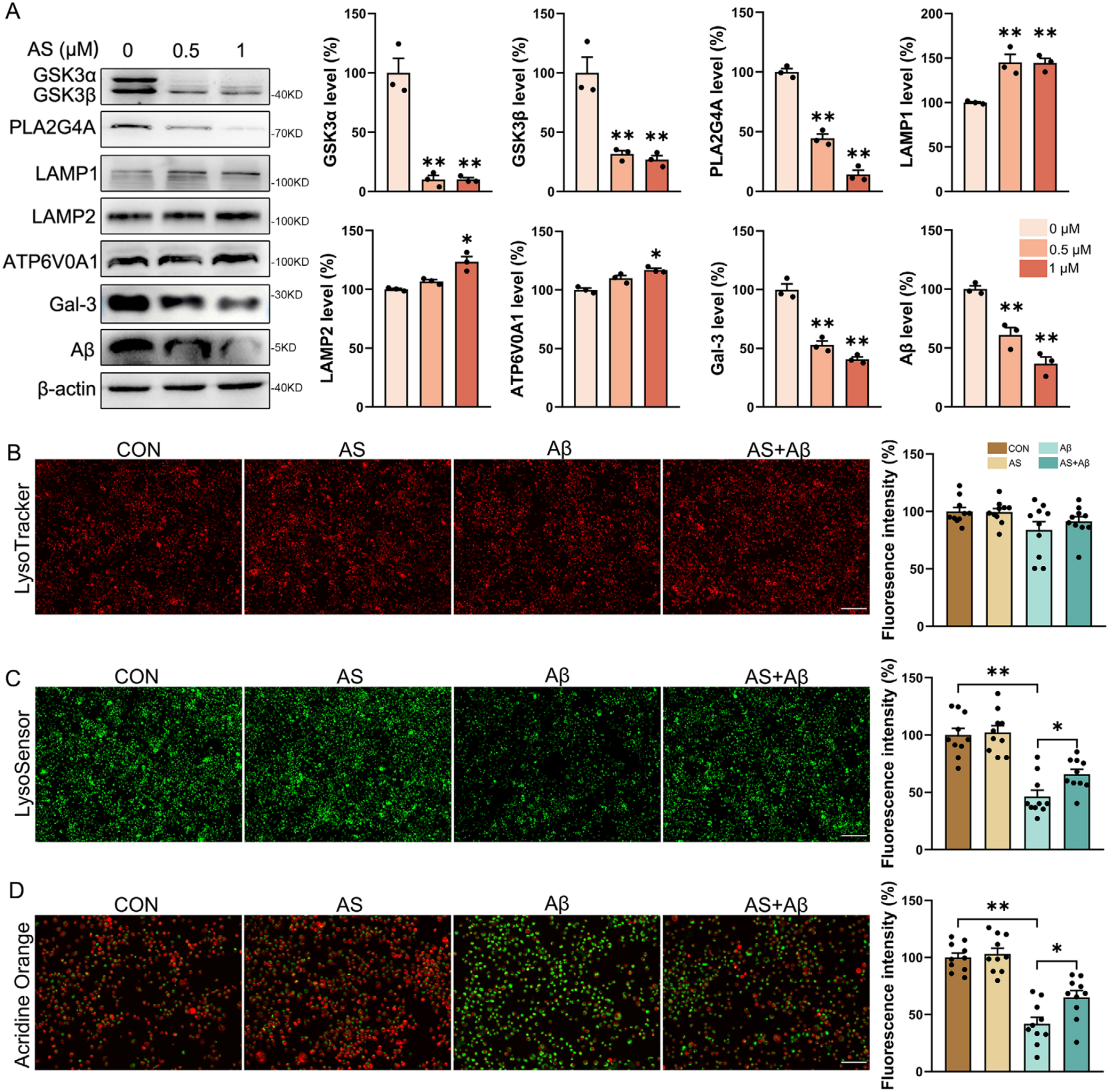
AS treatment reduces PLA2G4A levels and ameliorates lysosomal impairment in vitro. (A) Immunoblotting evaluated the expression levels of GSK3*α*/*β*, LAMP1, LAMP2, ATP6V0A1, PLA2G4A, Gal‐3, and A*β* in N2a‐sw cells treated with different concentrations of AS (0, 0.5, 1 μM) for 24 h. *n* = 3. (B–D) N2a cells were treated with A*β* oligomers (1 μM) and AS (0.5 μM) for 24 h before LysoTracker, LysoSensor, and AO staining of the cells. *n* = 10; Scale bar (LysoTracker and LysoSensor) = 200 μm; Scale bar (AO) = 100 μm. *p < 0.05, **p < 0.01.

To further investigate the role of AS in enhancing lysosomal function, we treated N2a‐sw cells with varying doses of AS. Consistent with our in vivo findings, AS treatment significantly reduced the expression of GSK3*α*/*β* (*p* < 0.01) and PLA2G4A (*p* < 0.01), while increasing the levels of LAMP1 (*p* < 0.01), LAMP2 (*p* < 0.05), and ATP6V0A1 (*p* < 0.05) in N2a‐sw cells. These changes were accompanied by decreased A*β* and Gal‐3 levels (*p* < 0.01, Figure 5A), suggesting that AS promotes A*β* degradation by downregulating PLA2G4A and thereby improving lysosomal function.

To determine whether AS directly ameliorates A*β*‐induced lysosomal dysfunction, we treated N2a cells with AS and/or A*β* oligomers and assessed lysosomal properties using three distinct probes (Figure 5B–D). AS treatment did not significantly alter LysoTracker fluorescence intensity, a measure of lysosomal content, in any of the treatment groups (Figure 5B *p* > 0.05). However, AS markedly attenuated the reduction in LysoSensor fluorescence, which reflects lysosomal acidification, induced by A*β* oligomers (Figure 5C *p* < 0.05). We further assessed lysosomal membrane integrity using Acridine Orange (AO), which accumulates in lysosomes and emits red fluorescence upon protonation; a decrease in the red/green fluorescence ratio indicates lysosomal membrane damage. AS treatment significantly alleviated the A*β*‐induced decline in the red/green fluorescence ratio (Figure 5D *p* < 0.05). Collectively, these results demonstrate that AS treatment mitigates A*β*‐induced lysosomal damage, likely through the downregulation of PLA2G4A.

### 3.6 AS Treatment Enhances A*β* Lysosomal Degradation via Inhibiting GSK3*β* /NF‐κB/PLA2G4A Signaling Pathway

**FIGURE 6:**
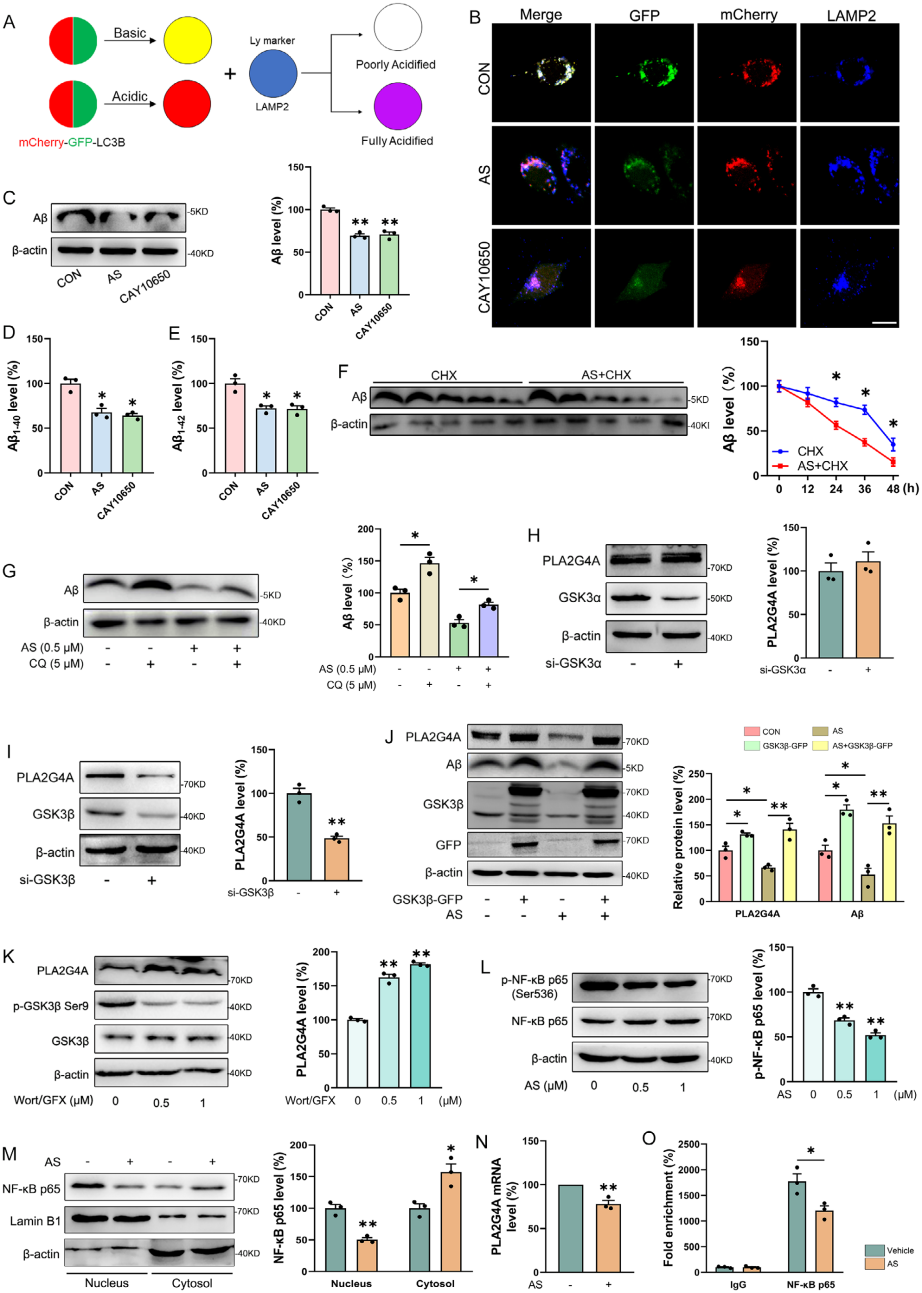
AS treatment enhances A*β* lysosomal degradation via inhibiting GSK3*β*/NF‐κB/PLA2G4A signaling pathway. (A) The schematic diagram of the fluorescence color development of the experiment. (B) Immunofluorescence analysis of mCherry (red), GFP (green), and LAMP2 (blue) in mCherry‐GFP‐LC3 transfected N2a‐sw cells treated with AS or CAY10650. Scale bar = 20 μm. The levels of A*β* in N2a‐sw cell lysates (C) and contents of A*β*_1‐40_ (D) and A*β*_1‐42_ (E) in the culture medium after treatment with AS (0.5 μM) or CAY10650 (0.5 μM) for 24 h. *n* = 3. (F) Immunoblotting evaluated the levels of A*β* in N2a‐sw cells after treatment with AS (0.5 μM) and/or CHX (20 μM) for 0, 12, 24, 36 and 48 h. *n* = 3. (G) Co‐treatment with CQ (10 μM) abolished AS (0.5 μM) ‐induced A*β* reduction in N2a‐sw cells. *n* = 3. (H‐I) The expressions of PLA2G4A after GSK3*α* (H) or GSK3*β* (I) knockdown in HEK293T cells. *n* = 3. (J) Levels of PLA2G4A and A*β* in N2a‐sw cells following treatment with AS and/or GSK3*β* overexpression. *n* = 3. (K) The expressions of PLA2G4A in N2a cells after treatment with different concentrations of Wort/GFX (0, 0.5, 1 μM) for 4 h. *n* = 3. (L) Expression of p‐NF‐κB in N2a cells treated with different concentrations of AS (0, 0.5, 1 μM) for 24 h. *n* = 3. (M) Immunoblotting evaluated the changes of NF‐κB levels in the nucleus and cytosol of N2a cells treated with AS (0.5 μM) for 24 h. *n* = 3. (N) The PLA2G4A mRNA levels in N2a cells treated with AS (0.5 μM) for 24 h. (O) ChIP‐qPCR results indicate that NF‐κB p65 directly binds to the PLA2G4A promoter, and this enrichment is reduced following AS treatment. *n* = 3. *p < 0.05, **p < 0.01.

Lysosomal function is critically dependent on the maintenance of luminal acidity. To assess the effect of AS on lysosomal acidification, we expressed mCherry‐GFP‐LC3B in N2a‐sw cells and labeled lysosomes with a blue marker, enabling discrimination between acidic (peach) and non‐acidic (white) lysosomes (Figure 6A). Both AS and the PLA2G4A‐specific inhibitor CAY10650 significantly ameliorated lysosomal acidification defects in N2a‐sw cells (Figure 6B). To further validate the role of PLA2G4A inhibition in A*β* clearance, we treated N2a‐sw cells with AS or CAY10650. Immunoblotting revealed that both treatments markedly reduced intracellular A*β* levels (Figure 6C *p* < 0.01), and ELISA assays showed significant decreases in secreted A*β*_1–40_ and A*β*_1–42_ levels in the culture medium (Figure 6D,E *p* < 0.05). Then, we performed CHX chase assays and found that A*β* decayed more rapidly in AS‐treated cells than in controls (Figure 6F *p* < 0.05). Moreover, co‐treatment with Chloroquine (CQ), a lysosomal function inhibitor, largely abolished the A*β*‐lowering effect of AS (Figure 6G *p* < 0.05). Collectively, these findings indicate that inhibition of PLA2G4A enhances lysosomal function and accelerates lysosomal clearance of A*β*.

To elucidate the mechanism by which AS inhibits PLA2G4A expression, we knocked down GSK3*α* or GSK3*β* (Figure 6H,I). PLA2G4A expression was markedly reduced upon GSK3*β* knockdown, but not GSK3*α* knockdown, indicating that PLA2G4A is specifically regulated by GSK3*β*. Conversely, overexpression (Figure 6J) or pharmacological activation (Figure 6K) of GSK3*β* upregulated PLA2G4A, confirming that GSK3*β* positively regulates PLA2G4A expression. Notably, GSK3*β* overexpression reversed the AS‐induced reductions in both PLA2G4A and A*β* levels (Figure 6J).

Given that NF‐κB is known to transcriptionally activate PLA2G4A [20, 29] and can be modulated by GSK3 [29, 30], we examined whether AS influences this pathway. AS treatment significantly reduced both the phosphorylation (Figure 6L *p* < 0.01) and nuclear translocation (Figure 6M *p* < 0.01) of NF‐κB, accompanied by a decrease in PLA2G4A mRNA levels (Figure 6N *p* < 0.01). ChIP‐qPCR further demonstrated that NF‐κB binds directly to the PLA2G4A promoter, and this binding was significantly diminished following AS treatment (Figure 6O *p* < 0.05). Collectively, these findings indicate that AS downregulates PLA2G4A expression through inhibition of the GSK3*β*/NF‐κB signaling axis, thereby restoring lysosomal function and promoting lysosomal degradation of A*β*.

## 4 Discussion

A substantial body of evidence indicates that aberrant overactivation of GSK3*α*/*β* serves as a critical mediator in driving A*β* deposition and Tau hyperphosphorylation [31, 32, 33]. Inhibiting GSK3*α*/*β* has been shown to significantly reduce both A*β* accumulation and the excessive phosphorylation and aggregation of Tau, leading to improved cognitive function in animal models of AD [16, 34, 35]. Thus, targeting GSK3*α*/*β* emerges as a promising therapeutic strategy for AD. In our previous research, we discovered that AS interacts with GSK3*α*/*β*, facilitating its exocytosis and subsequently lowering its intracellular levels. Then, we confirmed that AS can effectively cross the blood–brain barrier and inhibit Tau phosphorylation in the brains of P301S transgenic mice [23]. In the present study, we found that AS treatment downregulates GSK3*β* expression, which in turn suppresses the NF‐κB/PLA2G4A signaling pathway and subsequently promotes lysosomal degradation of A*β*. This cascade promotes lysosomal degradation of A*β*, ultimately leading to enhanced cognitive performance in APP/PS1 transgenic mice.

Lysosomal degradation of protein aggregates and damaged organelles is essential for cellular quality control [36, 37], preserving homeostasis and optimal function. Lysosomal activity depends on luminal acidity, soluble proteases, membrane integrity, and functional membrane proteins [38]. Among the numerous proteases in lysosomes [39], CTSD and CTSB are the most abundant and are highly expressed in neurons [40, 41]. In the brains of AD patients, lysosomes accumulate around A*β* plaques and within swollen axons [42], yet these lysosomes often exhibit impaired activity due to protease deficiencies [43]. Therefore, restoring lysosomal function represents a promising strategy to reduce A*β* accumulation [42]. Lysosomal acidity is closely linked to GSK3*α*/*β* activity; inhibition of GSK3*α*/*β* enhances lysosomal acidification and promotes substrate degradation [12]. Our findings may appear inconsistent with a recent study reporting that AS, as a forkhead box O1 (FOXO1) antagonist, abolished quercetin's beneficial effects in APP/PS1 mice [44]. However, that study examined AS co‐administered with quercetin rather than AS monotherapy. Mechanistically, we and others have demonstrated that AS reduces GSK3*α*/*β* independently of FOXO1 [22, 23], supporting the cognitive improvements observed in our study. These discrepancies likely reflect differences in experimental design, treatment duration, and disease stage. Future studies systematically comparing treatment windows will clarify the context‐dependent roles of FOXO1 and AS in AD pathology. Consistent with this, our study demonstrates that AS treatment reduces GSK3*α*/*β* levels in both APP/PS1 mouse brains and N2a‐sw cells, improves lysosome function, and decreases A*β* load. Moreover, AS treatment improved learning and memory in APP/PS1 mice. Collectively, these findings suggest that AS alleviates cognitive deficits by promoting lysosomal degradation of A*β* in the brain of APP/PS1 mice.

Phospholipase A2 (PLA2) enzymes catalyze the release of fatty acids from phospholipids [45]. Among these, PLA2G4A is a major phospholipase in the brain that hydrolyzes the sn‐2 acyl ester bond of phospholipids [18, 46]. Accumulating evidence indicates that PLA2G4A directly compromises membrane integrity, leading to increased lysosomal permeability and impaired lysosomal function [47, 48, 49]. In various neurodegenerative diseases, PLA2G4A activity and expression are markedly elevated [18, 19], and its activation promotes lysosomal membrane permeabilization and neuronal death. Conversely, inhibition of PLA2G4A has been shown to alleviate lysosomal damage [50]. In the present study, AS treatment reduced PLA2G4A expression in both APP/PS1 mouse brains and N2a‐sw cells. Moreover, treatment with AS or a PLA2G4A inhibitor enhanced lysosomal acidification. These findings suggest that AS improves lysosomal acidification and facilitates A*β* degradation, at least in part, by suppressing PLA2G4A expression.

PLA2G4A is transcriptionally upregulated by NF‐κB [20], and GSK3*β* activation promotes NF‐κB nuclear translocation [29, 30]. Given that AS downregulates both GSK3*α* and GSK3*β*, we examined their individual roles in regulating PLA2G4A. Knockdown of GSK3*β*, but not GSK3*α*, significantly reduced PLA2G4A expression. Furthermore, AS treatment suppressed NF‐κB phosphorylation and nuclear translocation, accompanied by decreased PLA2G4A transcription. Collectively, these results indicate that AS downregulates PLA2G4A expression through inhibition of the GSK3*β*/NF‐κB signaling axis.

## 5 Conclusion

In summary, our study demonstrates that AS treatment decreases intracellular GSK3*β* levels, thereby inhibiting the NF‐κB/PLA2G4A signaling pathway and promoting lysosomal acidification. This enhances lysosomal degradation of A*β*, ultimately reducing accumulation and ameliorating cognitive deficits in APP/PS1 mice. These findings highlight AS as a promising therapeutic candidate for AD.

## Author Contributions

Da‐Long He and Yong‐Gang Fan provided the study design; Da‐Long He and Zheng Wu performed most of the experiments and wrote the manuscript; Zheng Wu, Rong‐Jun Jia, Ting‐Yao Wu, and Yi‐Min Qiu contributed to experiments; Yong‐Gang Fan designed the experiments and edited the manuscript. All authors approved the final submitted version of the manuscript.

## Funding

This study was financially supported by the National Natural Science Foundation of China (82301626).

## Disclosure

Additional Supporting Informations and Methods can be found online in the Supporting Information section. The Supporting Information provides detailed descriptions of behavioral tests (open field, Morris water maze, novel object recognition), cell culture and treatments, A*β* oligomer preparation, lysosomal probe staining, and transfection protocols.

## Ethics Statement

This study was carried out according to the recommendations of “Laboratory Animals‐Guideline of Welfare and Ethics, The Ethics Committee for Medical Laboratory Animals of China Medical University” (IACUC: No. CMU2023516).

## Conflicts of Interest

The authors declare no conflicts of interest.

## Supporting information


**Data S1:** Supporting Information.

## Data Availability

The data that support the findings of this study are available from the corresponding author upon reasonable request.
