# Supplementary material for: AS1842856 Reduces β‐Amyloid Burden via Inhibiting PLA2G4A‐Mediated Lysosomal Dysfunction in APP/PS1 Mice
Source: CNS Neurosci Ther. 2026 Apr 28;32(4):e70910. doi: 10.1002/cns.70910 (PMC13123454; doi:10.1002/cns.70910)
Supplement: Supplementary file 1 — Data S1: Supporting Information. [file CNS-32-e70910-s001.docx]

**Supporting Information**

**Supplement Materials and Methods**

1 Behavioral tests

The trajectory and status of the mice were recorded using Smart 3.0 software, and all tests were performed in a blinded and randomized order.

1.1 Open field test

The open field test was conducted in a 40 × 40 × 40 cm box. The experimental environment was kept quiet, and the mice moved freely in the box for 5 min.

1.2 Morris water maze test

The Morris water maze test was conducted in a circular pool with a diameter of 1.5 m and a depth of 40 cm, in which milk was added to make the water opaque, and the pool was surrounded by an opaque enclosure to minimize external interference during the experiment. In the southwest (SW) quadrant of the pool, a circular platform with a diameter of 10 cm was placed. The visible platform period was the first 2 days, and the hidden platform period was the next 5 days. The time limit for mice to find the visible and hidden platforms was 1 min. In the visible platform period, the platform was set 1 cm above the water surface, and the mice entered the water from three different quadrants three times. If the mice could not find the platform within 1 min, they would be guided to the platform by the experimenter and stay on the platform for 30 s. In the hidden platform period, the platform was set 1 cm below the water surface, and the mice entered the water from three different quadrants as performed in the visible platform tests. On day 8 of the experiment, a probe trial was conducted during which the platform was removed, and the number of times mice crossed the original platform location within 1 min was recorded.

1.3 Novel object recognition

The novel object recognition test was conducted in a square box with a length, width, and height of 40 cm, and the test environment was kept quiet. Before the test, the mice were placed in the box for 10 min to adapt to the experimental environment. On the first day of the test, two identical objects were placed in symmetrical positions in the box, and the mice moved freely in the box for 10 min. The trajectories of the mice, as well as the times of recognizing the objects, were recorded. On the second day of the test, one of the objects was replaced by a new object with different shapes, and the mice moved freely in the box for 10 min. The trajectories and the times of the mice recognizing the two objects were recorded. After each mouse was finished, the box and the blocks were wiped clean to prevent the residual odor from affecting the subsequent experimental mice.

2 Cell culture and treatments

N2a cells, N2a-sw cells, and HEK293T cells were cultured in DMEM medium supplemented with 10% FBS and 1% penicillin/streptomycin. The culture environment was maintained at a constant temperature of 37 °C with 5% CO₂. Fresh medium was replaced every two days, and subculturing was performed when the cell density reached 80% confluence. The information on the compounds used in this study is as follows: AS (MCE, HY-100596); Chloroquine (CQ, MCE, HY-17589A); CAY10650 (MCE, HY-10801); Wortmannin (Wort, GLPBIO, GC12338); GF109203X (GFX, GLPBIO, GC15431); Cycloheximide (CHX, Caymanchem, 14126).

3 Aβ oligomer preparation

Aβ_1-42_ dry powder (ChinaPeptides) was dissolved in HFIP (Sigma, 440671) and dried under light protection until the HFIP evaporated. The peptides were subsequently re-dissolved in 1 × PBS containing 1% DMSO. And the solution was incubated at 4 °C for 24 h to achieve Aβ oligomers.

4 Lysosomal probes staining

Solvent control, AS (0.5 μM), Aβ (1 μM), and AS (0.5 μM) plus Aβ (1 μM) were added to the N2a cells for incubation for 24 h. The LysoTracker (Invitrogen, L7528, 50 nM), LysoSensor (Invitrogen, L7535, 1 μM), and Acridine Orange (AO, Solarbio, CA1143, 10 mg/mL) were stained for 30 min. Then, the image acquisition was immediately performed in an inverted fluorescence microscope. Images were quantitatively analyzed using Image J software.

5 Transfections

100 pM siRNA or 2 μg of plasmid per well was transfected using the Trans-geneverTM Transfection Reagent (GENEVER, B07001) following the manufacturer's instructions. The following plasmids were used: GSK3β cDNA ORF Clone, Human, N-GFPSpark® tag (Sino Biological, HG10044-ANG); pCMV-mCherry-GFP-LC3B (Beyotime Biotechnology, D2816). The siRNAs used in this paper: GSK3α, 5’-GAAAGACGAGCUUUACCUATT-3’; GSK3β, 5’-GCUCCAGAUCAUGAGAAAGCUTT-3’. After transfection, the cells were further cultured for 48 h for subsequent processing.
